# Supplementary material for: Time‑resolved multi-omic analysis of paclitaxel exposure in human iPSC‑derived sensory neurons unveils mechanisms of chemotherapy‑induced peripheral neuropathy
Source: Cell Death Dis. 2026 Feb 10;17(1):211. doi: 10.1038/s41419-026-08445-2 (PMC12921266; doi:10.1038/s41419-026-08445-2)

Linie 264a

n=17

(KGlu intracellular solution with Alexa488)

Em= -59.9 ± 6 mV
Rm= 162.7 ± 91 MOhm
AP halfwidth= 1.9 ± 0.6 ms
AP amplitude= 69.4 ± 16 mV
instantaneous frequency: 46 ± 17 Hz

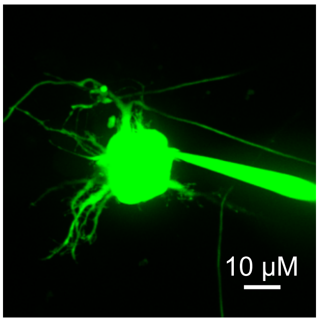

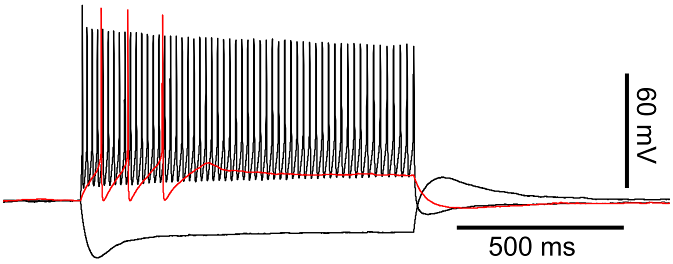

Line 264a 21_04_06 6th cell

Em= -66.3 mV
Rm= 60 MOhm

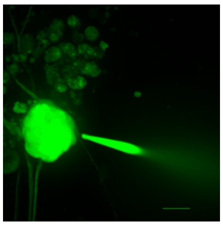
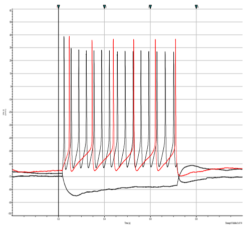
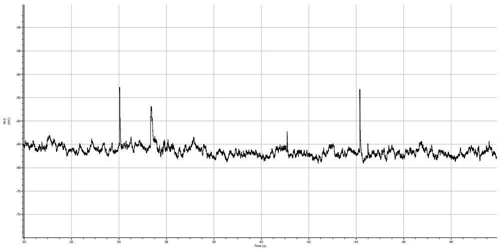

Line 264a 21_03_31 1. cell

Em= -65.8 mV
Rm= 153 MOhm

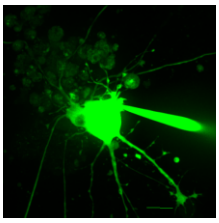
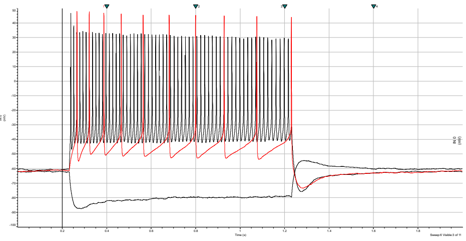
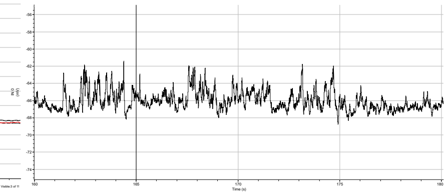

Line 264a 21_03_31 2nd cell

Em= -67 mV
Rm= 120 MOhm

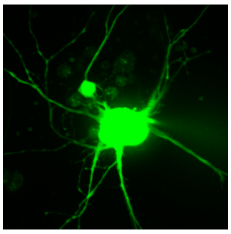
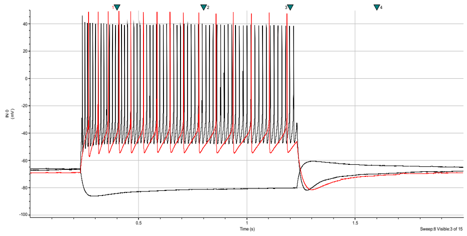
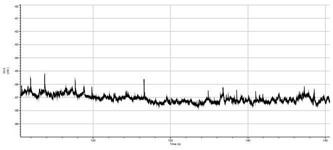
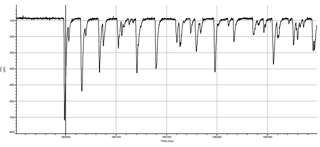

Line 264a 21_03_31 3rd cell

Em= -66.8 mV
Rm= 157 MOhm

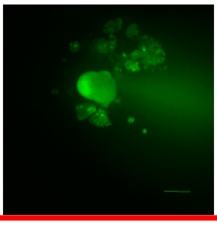
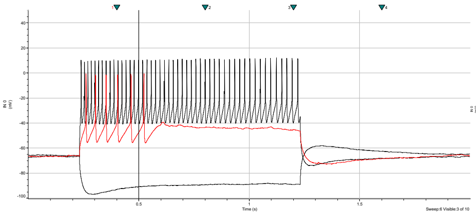
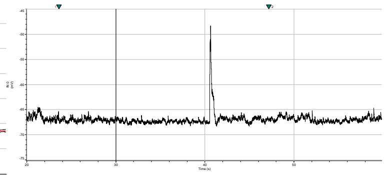

Line 264a 21_03_31 4th cell

Em= -63.9 mV
Rm= 80 MOhm

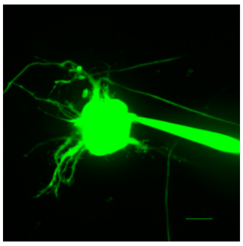
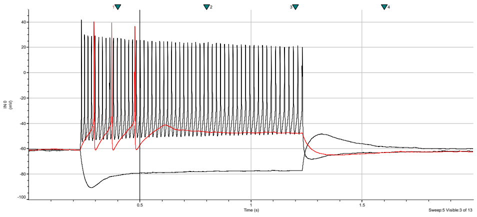
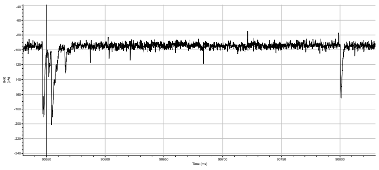

Line 264a 21_03_31 6th cell

Em= -64 mV
Rm= 156 MOhm

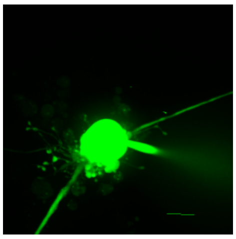
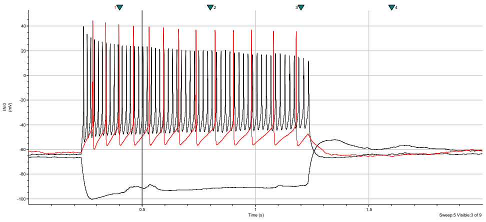
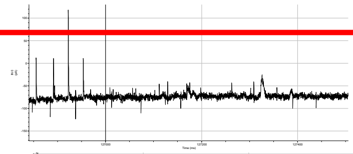
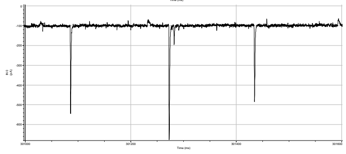

Line 264a 21_03_22 1 cell

Em= -62.3 mV
Rm= 333 MOhm

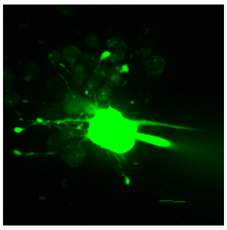

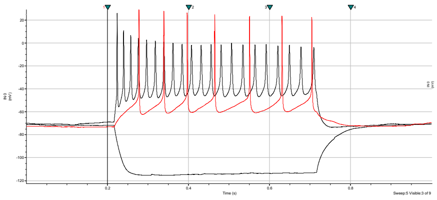

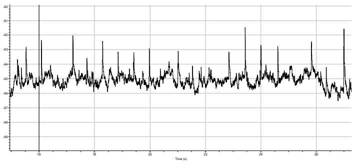

Line 264a 21_03_22 2nd cell

Em= -63 mV
Rm= 269 MOhm

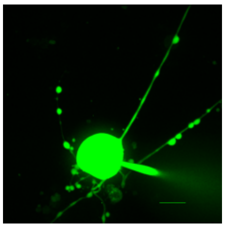

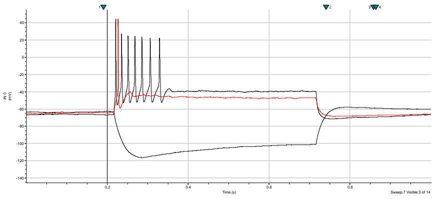

Line 264a 21_03_22 3. cell

Em= -58.6 mV
Rm= 344 MOhm

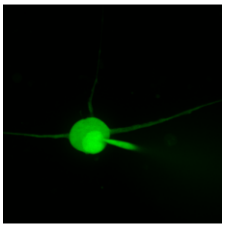

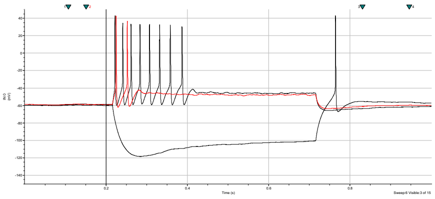

Line 264a 21_03_22 4. cell

Em= -58 mV
Rm= 211 MOhm

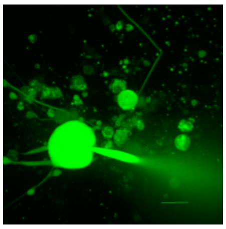

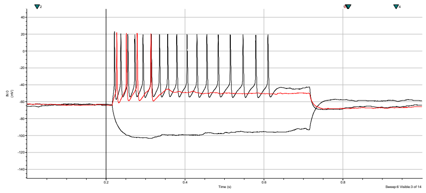

Line 264a 21_03_22 5. cell

Em= -60 mV
Rm= 700 MOhm !!

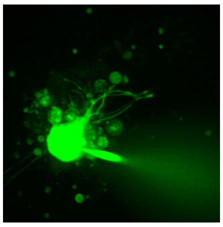

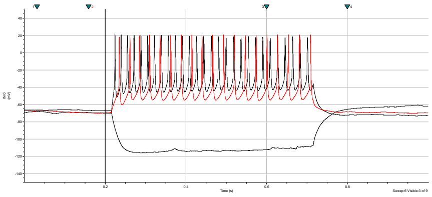

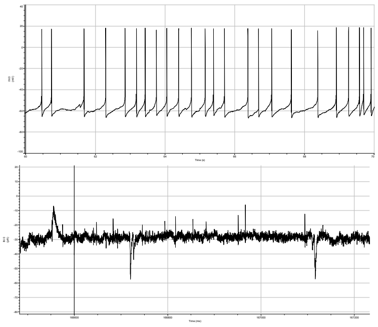

Line 264a 21_03_22 7. cell

Em= -60 mV
Rm= 700 MOhm !!

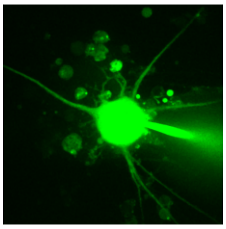

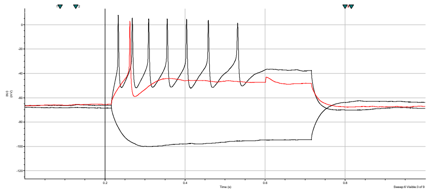

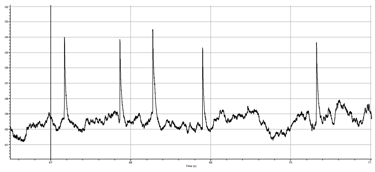

Supplement: Supplementary file 15 — Dataset 1. [file 41419_2026_8445_MOESM15_ESM.zip › CDD_Open_Data/07_Electrophysiology/Linie264a.pdf]
